# Grapevine Yield and Leaf Area Estimation Using Supervised Classification Methodology on RGB Images Taken under Field Conditions

**DOI:** 10.3390/s121216988

**Published:** 2012-12-12

**Authors:** Maria-Paz Diago, Christian Correa, Borja Millán, Pilar Barreiro, Constantino Valero, Javier Tardaguila

**Affiliations:** 1 Instituto de Ciencias de la Vid y del Vino (CSIC, University of La Rioja, La Rioja Government) Madre de Dios, 51, 26006 Logroño, Spain; E-Mails: borja.millanp@unirioja.es (B.M.); javier.tardaguila@unirioja.es (J.T.); 2 Department of Agricultural Engineering, ETSIA, Technical University of Madrid, Av. Complutense s/n Ciudad Universitaria, 28043 Madrid, Spain; E-Mails: ccorrea@udec.cl (C.C.); pilar.barreiro@upm.es (P.B.); constantino.valero@upm.es (C.V.)

**Keywords:** clustering, Mahalanobis, *Vitis vinifera* L., vineyard, yield assessment

## Abstract

The aim of this research was to implement a methodology through the generation of a supervised classifier based on the Mahalanobis distance to characterize the grapevine canopy and assess leaf area and yield using RGB images. The method automatically processes sets of images, and calculates the areas (number of pixels) corresponding to seven different classes (Grapes, Wood, Background, and four classes of Leaf, of increasing leaf age). Each one is initialized by the user, who selects a set of representative pixels for every class in order to induce the clustering around them. The proposed methodology was evaluated with 70 grapevine (*V. vinifera* L. cv. Tempranillo) images, acquired in a commercial vineyard located in La Rioja (Spain), after several defoliation and de-fruiting events on 10 vines, with a conventional RGB camera and no artificial illumination. The segmentation results showed a performance of 92% for leaves and 98% for clusters, and allowed to assess the grapevine’s leaf area and yield with R^2^ values of 0.81 (*p* < 0.001) and 0.73 (*p* = 0.002), respectively. This methodology, which operates with a simple image acquisition setup and guarantees the right number and kind of pixel classes, has shown to be suitable and robust enough to provide valuable information for vineyard management.

## 1. Introduction

The great economic, social and environmental importance of the viticulture and wine industry worldwide encourages the development and application of innovative technologies aimed at objective monitoring vineyards to improve grape and wine quality.

One of the historical main goals of the wine industry has been the accurate and objective estimation of the yield [1–3] and of the vineyard’s winegrape quality potential. More specifically, *yield forecasting* has been identified in recent years as one of the more profitable topics for scientific research in viticulture [4] as it could lead to more efficiently managed vineyards producing wines of better quality [5]. So far, most of the methods employed for yield estimation are destructive, labour and time demanding [6], or very expensive [7]. Similarly, the assessment of a vineyard’s winegrape quality potential has often been attempted by the use of vineyard score sheets [8–12] which required visual evaluation of several grapevine canopy variables, such as vigour, leaf status, exposed leaf area, canopy porosity and fruit exposure, all of them intrinsically related to final grape and wine composition and quality [13–19]. Consequently, there is a need for assessing the vineyard yield and winegrape quality potential by objective monitoring the grapevine canopy features, but customary methods for obtaining canopy measurements, such as the Point Quadrate [8] or LIDAR [20–22], though quantitative, are limited in their precision and practicality, either because they are time-consuming or expensive. Hence, new methods are required to assess grapevine canopy status, and image capturing and analysis may be an objective and potentially useful technique to replace time-consuming procedures and to provide useful information for more efficient grapevine canopy management.

In recent years several studies, based on image processing, have been conducted in order to assess features of the vineyard canopies, like in [23–25] for general purposes and also for specific applications like disease detection [26], smart spraying [27,28] and yield estimation [29]. These studies were carried out in order to quantify features such as leaves, vine shoots, trunks and grapes. However these investigations required sophisticated equipment and specialized software for analysis and interpretation. A simpler layout for image capturing and processing for the assessment of grapevine canopy features was described in the works of Dunn and Martin [1], who estimated the yield, and of Tardaguila *et al.*[30,31]. In these works digital image analysis techniques applied to sample data from a defoliation study revealed quantitative descriptions of canopy biomass distribution, fruit exposure, cluster compactness, and treatment efficacy, although the image processing was not completely automated.

Colour classification techniques in the Red Green and Blue (RGB) colour space can be divided into supervised and unsupervised [32]. In supervised methods, the number of classes is specified and the supervisor selects the prototype of these classes. Conversely, in unsupervised methods, the characteristics of the classes are unknown, and the classification algorithm ascribes membership in such a way that the elements in each class will exhibit similar characteristics and are more similar to each other, than with respect to elements of other classes. Supervised and unsupervised methods have been used outdoors [33] and specifically for vineyard feature extraction aiming at vigour characterization [34]; grape clusters and foliage [27]; single grapes [35]; count ‘fruit pixels’ for yield estimation [1], or segregate grapes, leaves and shoots [36,37].

In unstructured environments, such as an agricultural field, conditions are variable, so robustness of unsupervised algorithms may be at risk [32]. Therefore supervised classification techniques are of special interest in this field, since a training set can be prepared by *a priori* establishing what features will correspond to the elements of a class [38], which, in turn, reduces uncertainty and leads to the possible solutions.

Our work aims to develop a fast, robust and inexpensive methodology for straightforward RGB image processing and interpretation, using images taken in the field, for grapevine canopy feature extraction that would enable accurate leaf area and yield estimation.

## 2. Experimental Section

To be able to correlate the estimated leaf area and yield data with real plant measurements, a detailed experimental setup for the acquisition of images was developed, based on successive defoliations and cluster thinning steps of individual vines.

### 2.1. Experimental Site

The experiments were conducted in 2010 in a commercial dry-farmed cv. Tempranillo (*Vitis vinifera* L.) vineyard, located in Casas Blancas, Cidamón (lat. 42°29′8.83″ N; long. 2°50′22.57″ W; 181 m asl, La Rioja, Spain). Tempranillo vines were grafted onto 41B rootstock and planted in 2005 following a between-row and within-row spacing of 2.70 m × 1.15 m respectively. The vines were spur-pruned (12 buds per vine) on a bilateral cordon and trained to a VSP trellis system. The trellis featured a supporting wire at 0.70 m, two wires at 1.00 m aboveground for protection against wind damage, and a pair of movable shoot-positioned wires at 1.45 m.

### 2.2. Defoliation, Cluster Thinning and Assessment of Removed Leaf Area and Fruit

In order to provide a good validation of the images’ classification method, at harvest (30 September 2010), 10 vines were randomly chosen, and each of them was individually and successively defoliated and cluster thinned in several steps as shown in Table 1. After each step, the leaf area and/or fruit removed were also recorded. This way, a range of different conditions of leaf area and cluster exposure were created to provide a better validation of the image analysis methodology.

The whole canopy of each vine was successively defoliated: first by removing the first six main basal leaves (step 1), then other six (in total 12 leaves) main basal leaves (step 3), and then the remaining main leaves and laterals (complete defoliation, or step 5). The number of leaves removed at each step was recorded and measured using a leaf area meter (LI-3100C; Li-Cor, Lincoln, NE, USA). Similarly, the whole canopy of each vine was successively de-fruited by thinning some clusters: first by removing every third cluster (step 2), then every second remaining cluster (step 4) and then the remaining clusters (step 6). The number of clusters removed and their combined weight was recorded after each cluster thinning event.

### 2.3. Image Acquisition

Before any defoliation or cluster thinning, and after each canopy manipulation step, each vine (10 vines in total) was photographed with a conventional RGB camera (Pentax model K200D, Tokio, Japan) mounted on a tripod set normal to the canopy 2 m from row axis and 1.05 m aboveground. Note that, when the defoliation process was performed over highly dense canopies, the distance to the remnant foliage increased and consequently the objects size seemed to be reduced. In order to correct this problem, images were scaled to fit the images acquired at 2 m. In this way all images represented the same area. A white screen was placed behind the canopy to avoid confounding effects from background vegetation and no artificial illumination was employed. Images were captured at a resolution 3,504 × 2,336 and reduced to 800 × 600 in order to speed up processing time. For each individual vine a total number of seven images were taken (Table 1).

### 2.4. Image Processing (Clustering Algorithm)

Several measurements of similarity between groups in terms of multiple characteristics have been proposed in the literature [37], but the Mahalanobis distance has been found to be the most suitable in a majority of applications, and it is widely used for pattern recognition and data analysis [39]. It is now known that many standard distance measurements such as Kolmogorov’s variational distance, the Hellinger distance, Rao’s distance, *etc.* are increasing functions of Mahalanobis distance under assumptions of normality and homoscedasticity [40].

Mahalanobis measures the similarity between an unknown sample group and a known one; it takes into account the correlations of the data set, and it is scale-invariant. It also accounts for the fact that the variances in each direction are different as well as for the covariance between variables.

The Mahalanobis distance between two random vectors $\left(\vec{x},\vec{y}\right)$ with the same distribution, and covariance matrix *S*, can be defined as:

(1) $$d\left(\vec{x},\vec{y}\right)=\sqrt{{\left(\vec{x}-\vec{y}\right)}^{T}S^{-1}\left(\vec{x}-\vec{y}\right)}$$

The Mahalanobis colour distance standardizes the influence of the distribution of each feature considering the correlation between each pair of terms [41].

In the case of RGB colour images *S* is computed as:

(2) $$S=\left[\begin{array}{ccc} \sigma_{\mathrm{RR}} & \sigma_{\mathrm{RG}} & \sigma_{\mathrm{RB}}\\ \sigma_{\mathrm{GR}} & \sigma_{\mathrm{GG}} & \sigma_{\mathrm{GB}}\\ \sigma_{\mathrm{BR}} & \sigma_{\mathrm{BG}} & \sigma_{\mathrm{BB}} \end{array}\right]$$

and the elements of S can be calculated as:

(3) $$\sigma_{\mathrm{RG}}=\sigma_{\mathrm{GR}}=\frac{1}{n-1}\sum_{i=0}^{n}\left(\mathrm{Ri}-\bar{R}\right)\left(\mathrm{Gi}-\bar{G}\right)$$

where R_i_,G_i_,B_i_ are the values of the i^th^ match (I = 1,2,3,...n), and *R̄*, *Ḡ*, *B̄* are the mean color values for R, G, and B in the given image, respectively.

For our purposes x⃗ was a three dimensional vector (R, G, B), that represented pixels from the image to be processed and y⃗ was also, a three dimensional vector (R, G, B), that represented the reference pixels (reference group) for each class to be identified.

The effect of S was to scale the distance along each feature. For image processing purposes, each channel R, G, B was considered as a feature.

**Figure 1.**
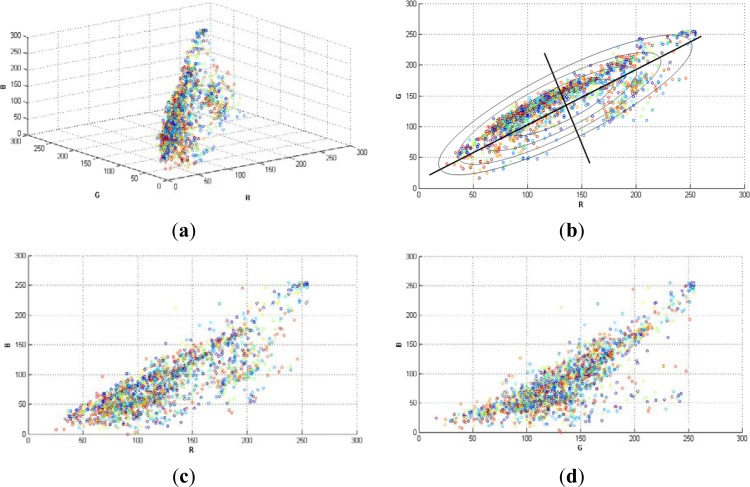
(**a**) Random selection of 2,000 pixels in a typical grapevine image (image I0 in RGB colour space). (**b**) RG plane. Ellipses represent equidistant points. (**c**) RB plane. (**d**) GB plane.

Figure 1, shows a random selection of 2,000 pixels in a typical grapevine image (RGB colour space). Note that, the variances in each direction (R, G, B) were different. This is the reason why it was necessary to implement a classifier that considered such differences, like Mahalanobis does. The region of constant Mahalanobis distance around the mean formed an ellipse in 2D space as in Figure 1(b).

### 2.5. Software Implementation

In the proposed methodology of this work seven reference groups of pixels were selected in order to generate the classification, in which every group represented a relevant characteristic of the grapevine canopy. The seven classes identified were: Young leaves (two classes), Old leaves (two classes), Wood (including shoots and trunk), Grapes and Background (or canopy gaps). However, if any of these classes was not present (depending on the growing stage), or a new class appeared on the image, the number and/or the group labels could be modified.

In this way, each pixel group was manually selected from a set of 10 representative images. For every pixel reference group a set of 40 pixels was chosen. Once the reference pixels were selected, the Mahalanobis distance was computed over a set of 70 images and its pixels were assigned to the class with the lowest distance. Of the total pool of 70 images (seven images per vine), images I0, I2 and I4 were used to train the model for the estimation of leaf area, as these images corresponded to the defoliation events. Similarly, I1, I3 and I5 were used to build the model for yield estimation.

**Figure 2.**
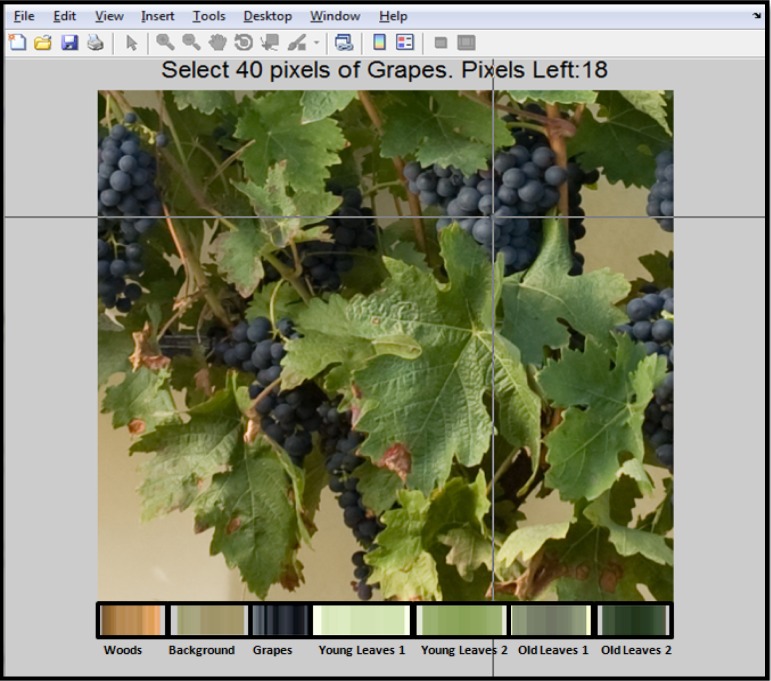
Screenshot of the manual selection of reference pixels.

In order to implement the classification algorithm and to provide graphical interface to the user, software based on Matlab 7.11 was developed. Figure 2 shows a software screenshot, at the moment when reference pixels are manually selected and depicted as a set of bars in the lower part of the image. These bars are displayed to bring visual feedback regarding the pixels selected as reference.

The number of pixels used as reference, as well the name and number of clusters can be modified by the users, in order to adjust the algorithm to different conditions, such as illumination changes or new characteristics that had not been previously observed, for example leaf colouration/discolouration produced by diseases.

#### 2.5.1. Feature Extraction Methodology Steps

The implemented methodology consisted on twelve steps which can be summarized as follows:

Step 1 Selection of representative images, containing as much variability (inside each class) as possible, and reading of reference images.
Step 2 Selection of 40 reference pixels for each class of interest.
Step 3 Reading from the directory containing the images to be processed.
Step 4 Processing of a section of the image (region of interest, ROI) or the whole image. Selection of the ROI if applicable.
Step 5 For each class, computation of the Mahalanobis distance between the reference pixels and the image/section to be analyzed.
Step 6 Assignation to class membership based on the rule that minimum distance from pixel to class reference pixels drives the allocation in a given class.
Step 7 Performance of morphological operations over the Grape class. Removal of small pixels groups and filling “holes” inside the Grape cluster by using erode and dilate morphological operations, respectively.
Step 8 Allocation of pixels to the Grape class only if they corresponded to the lower half of the image. e.g., If the image resolution was 800 × 600, the pixels to be considered as valid for the Grape class must be within the 400 to 800 position of the vertical axis.
Step 9 Computation of the number of pixels for each class.
Step 10 Saving the numerical results on a spreadsheet.
Step 11 Saving the class images in a directory.
Step 12 Displaying the class images on screen.

### 2.6. Algorithm Validation

A validation process for these specific grapevine canopy images was carried out. This validation was manually performed, selecting some ROIs on images that showed representative conditions of illumination and colours. Once the ROI was selected, the number of pixels for each class was manually counted by an expert, both on the original and the clustered image.

### 2.7. Correlations. Leaf Area and Yield Estimation

For leaf area and yield estimation, the set of images was divided into two groups: the training group, in which two thirds of data were used to generate the model, and the validation group, where the remaining one third of data was allocated for validation purposes. For the training group, linear correlations were run between the number of pixels of the Leaves and Grape classes in each image, and the actual leaf area and yield present in the vine at that time, respectively (SPSS v15.0, IBM, Armonk, NY, USA). Hence, these correlations were used to estimate leaf area and yield in the set of images of the validation group, and correlations between the estimated and observed (real) values for leaf area and yield were run, and the coefficients of determination (R^2^) and root mean squared error (RMSE) were computed.

## 3. Results and Discussion

### 3.1. Algorithm Validation

**Figure 3.**
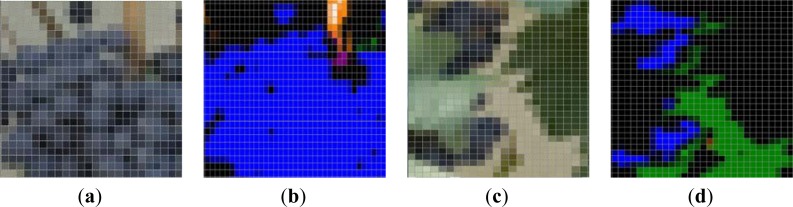
Manual validation of classification. (**a**) Original image, where 73.1% are Grape pixels. (**b**) Clustered image. Blue pixels correspond to the Grape class, and represented 72% of the image. (**c**) Original image, where 58% are Leaf’s pixels. (**d**) Clustered image. Black pixels correspond to leaves, and represented 63% of the image.

Examples of the ROI (30 × 30 pixels) selected for the manual validation process of the algorithm are depicted on Figure 3. The manual validation showed a 98% of correct classification for the Grape class and a 92% for the Leaves (Young and Old leaves groups added). Most of the misclassifications in the Leaves’ groups were due to younger shoots and laterals, which exhibited almost the same green colour than leaves.

**Figure 4.**
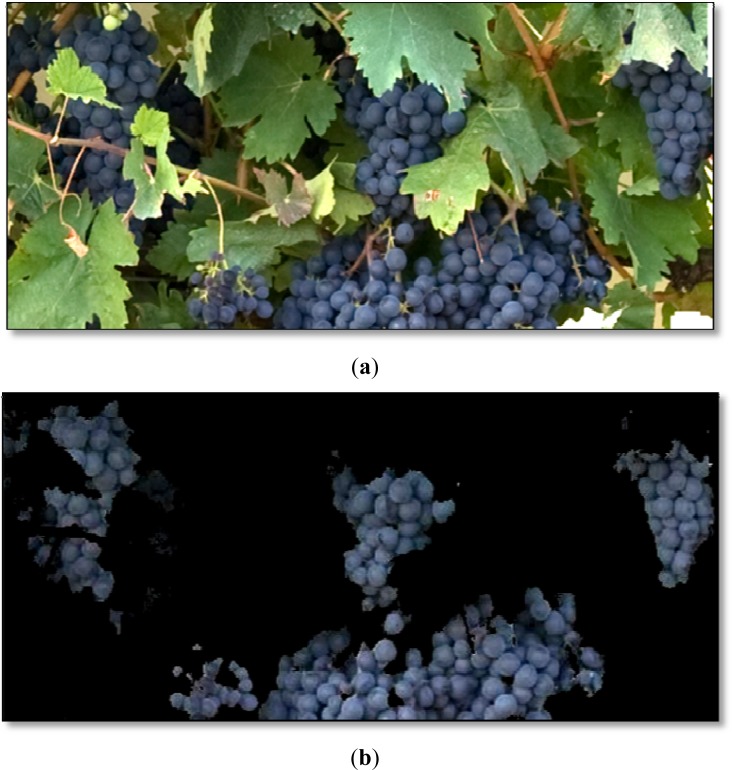
(**a**) Grapes class detail in the original image. (**b**) Detail of the identified grapes before the morphological operations.

As the Figure 4(a,b) and the manual validation process show, the classifiers performed very well without any image pre-treatment, such as improvement of contrast, brightness or colour adjustment. This is an important outcome, which makes the process simpler, compared to previous works where images had to be cut, reoriented or strongly pre-processed [42], and especially interesting, given the fact that no artificial illumination was used for the image acquisition in the vineyard.

### 3.2. Classifier Performance

**Figure 5.**
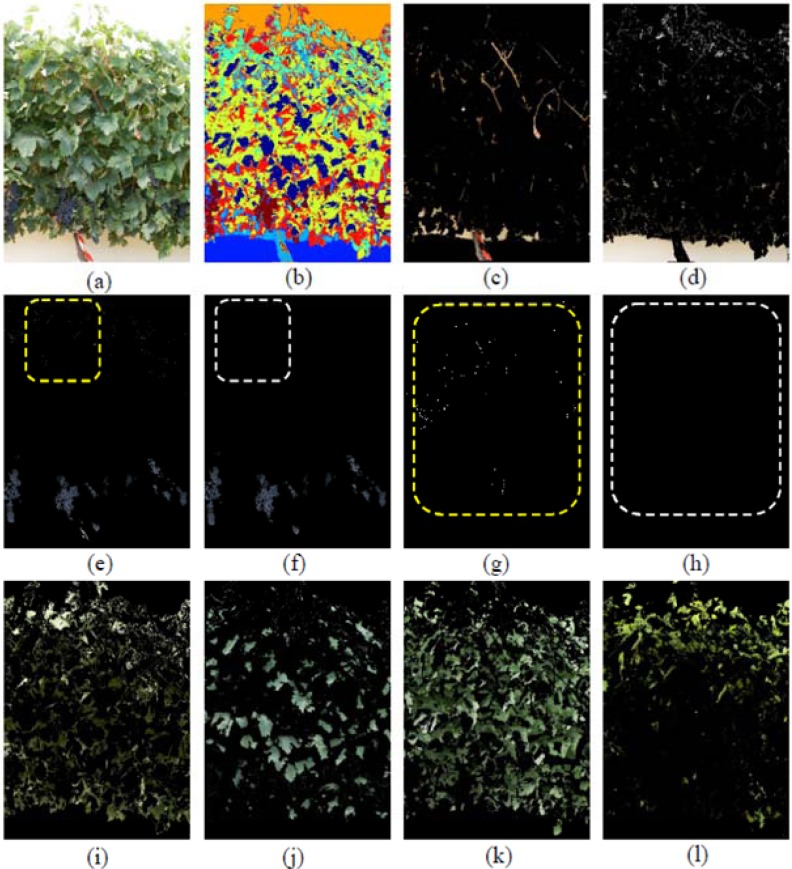
Classification example performed over a grapevine image of a non-defoliated, non-thinned vine (Image I0). (**a**) Original image. (**b**) Clustered image. (**c**) Vine wood. (**d**) Background. (**e**) Grape class, without morphological operations. (**f**) Grape class after morphological operations. (**g**) Zoomed area in part (**e**) showing small pixel groups misclassified. (**h**) Zoomed area in part (f) showing how the morphological operations removed the small pixel groups. (**i**) Old leaves grade 1. (**j**) Old leaves grade 2. (**k**) Young leaves grade 1. (**l**) Young leaves grade 2.

**Figure 6.**
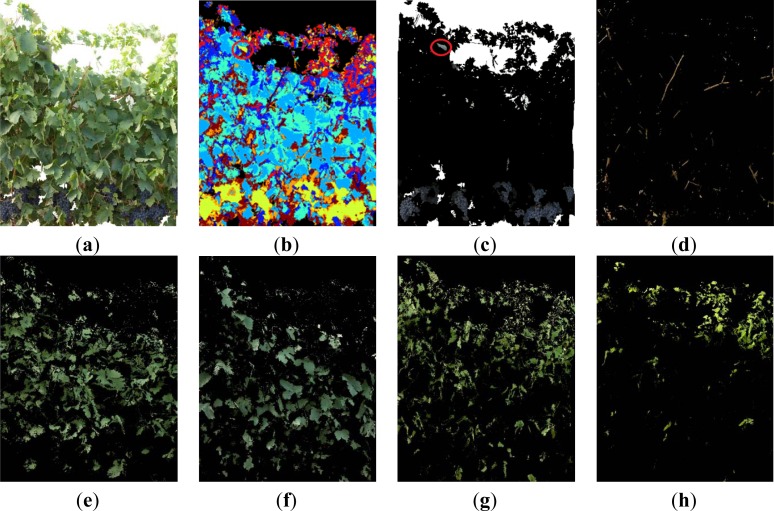
Classification example, performed over a grapevine image after the first defoliation step (I1) to show the algorithm capability to separate between different kinds of leaves (**a**) Original image. (**b**) Clustered image. (**c**) Grape class, without morphological operations. (**d**) Wood. (**e**) Old leaves grade 1. (**f**) Old leaves grade 2. (**g**) Young leaves grade 1. (**h**) Young leaves grade 2.

**Figure 7.**
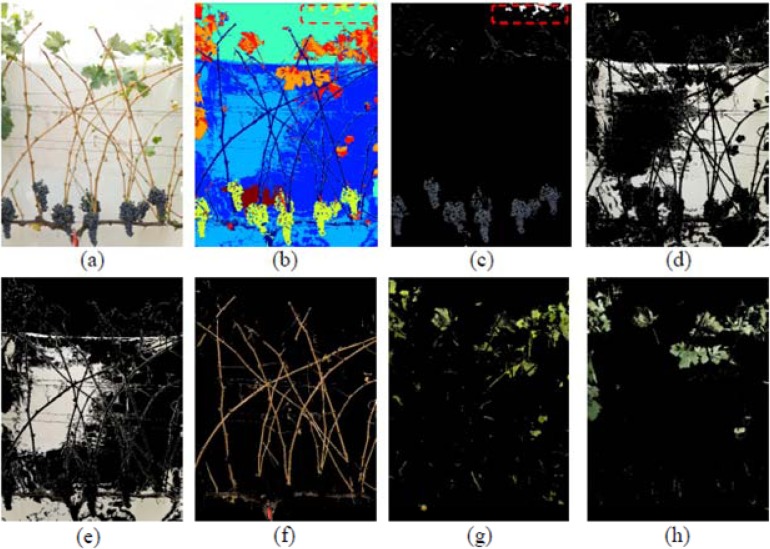
Classification example performed over a grapevine image after the third defoliation stage (Image I5). (**a**) Original image. (**b**) Clustered image. (**c**) Grape class. (**d**) Bright background. (**e**) Dark background. (**f**) Vine wood. (**g**) Young leaves. (**h**) Old leaves.

In order to illustrate the classifier’s performance, three images that were representative of the presence of misclassification errors were selected for detailing all generated classes. These images corresponded to three different defoliation stages and are presented in Figures 5–7. Likewise, in Figure 5, a non-defoliated, non-thinned grapevine image at step 0 illustrating Grape pixels misclassification errors was chosen.

Then, two images showing other common misclassification errors such as atypical leaf colouration (Figure 6) or the influence of the sky (Figure 7) were selected.

In Figure 5 seven classes were generated: Grapes, Wood (shoots and trunk), Background, and four different types of Leaves, which included Young leaves grade 1, Young leaves grade 2, Old leaves grade 1 and Old leaves grade 2, being the numbering of grades equivalent to increasing stages of leaves’ maturity. The differentiation of several kinds of leaves (Figure 5(I–l)) has important implications if a visual assessment of the vine physiology performance is intended, as younger leaves are photosynthetically more active than older leaves [43] and reveal differences in the physiological behaviour of the plant, which may alert of the presence of abiotic stresses such as water or nutrient deficiencies. Grape class was wrongly estimated at a first stage (Figure 5(e)) due to two combined effects. First, the Grape regions also included small white bright pixels wrongly assigned to the Background class (Figure 5(d)), which actually corresponded to the waxy-white coating that is visible in grapes, and known as bloom, leading to underestimation of the Grape class. On the other hand, bluish pixels on the top of the image corresponding to some leaves’ areas (Figure 5(e), and detail in part (g)) were misclassified into the Grape class, with its subsequent overestimation. These errors were drastically reduced when these small pixel groups were deleted by a set of morphological operations. Specifically, erode and dilate consecutive operations over the Grape class were performed to eliminate these pixels as depicted on the Figure 5(f) (detailed in (h)).

When the first defoliation step was performed (image I1), more clusters were exposed, the lighting conditions over the grapes improved, and consequently, their detection rate increased (Figure 6(a,b)). In Figure 6b, the Grape class appears in yellow, Wood class in dark blue, and the four classes of leaves (Young leaves grade 1, Young leaves grade 2, Old leaves grade 1, Old leaves grade 2), are presented in blue, light blue, cyan and red, respectively. When compared with the four Leaf classes shown in Figure 5, leaves in Figure 6 (classes (e) (f) (g) and (h)) were similar, showing that the classification algorithm was robust at segregating several foliar maturity stages when lighting conditions changed and defoliation was conducted.

In this image (I1), the Grape class also showed a misclassification event, as in the left upper corner, enclosed in a red circle (Figure 6(b,c)) some leaves with a blue colouration induced by the spraying of a fungicide (copper sulphate) were confounded and considered as Grape pixels. In this case, the misclassification was not caused by a poor performance of the classification methodology, but by the hue similarity between the sprayed leaves and the Grape class. In this scenario, and to improve the global algorithm performance, pixels were classified as Grape pixels, only if they were located at the bottom half of the image. Also in the case of images with low variability of leaf types and adverse sunlight conditions, the algorithm showed adequate response and adaptability (Figure 7).

After the final defoliation stage, only the vine shoots and remaining clusters were visible on the grapevine canopy (image I5, Figure 7(a)). Under these conditions, and as the background could not cover the entire area, the sky could be partially distinguishable, and the sunlight penetrated into the image scene creating shadows and bright areas over the background. To overcome this situation, two different classes of background were selected, and identified as Dark and Bright Background classes (Figure 7(d,e)) and the four classes of leaves described and identified in previous steps, were reduced to only two clusters: Old and Young leaves (Figure 7(g,h)). The complete classification performance is shown in Figure 7(b).

Depicted on Figure 7(f), the Wood class included the vine trunk, shoots, and trellis wires. On the other hand, Figure 7(c) shows the Grape class, with preliminary misclassifications, enclosed in red in the upper part of the image, due to some pixels of blue colouration corresponding to the sky. This misclassification was also solved by considering as grape pixels only those located at the lower half of the image.

### 3.3. Grape Yield and Leaf Area Estimation

**Figure 8.**
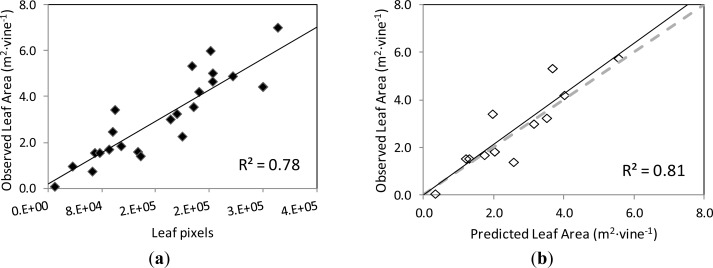
(**a**) Correlation between the actual leaf area (m^2^·vine^−1^) of the grapevine canopy in the images of the training set, and the number of pixels corresponding to the Leaf class computed by the classification methodology and image analysis. (**b**) Comparison between the actual values of leaf area (m^2^·vine^−1^) of the grapevine canopy in the images of the validation set, and the predicted leaf area values calculated with the correlation equation in (a). Dotted line is 1:1 line.

**Figure 9.**
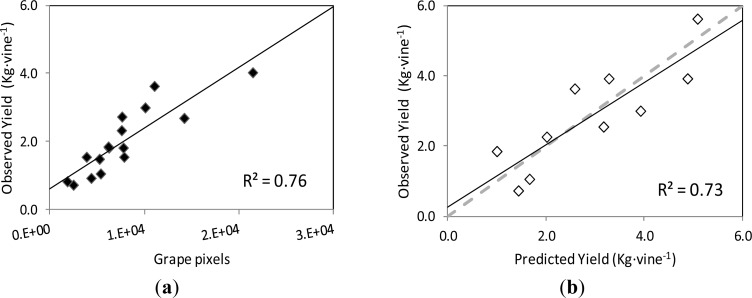
(**a**) Correlation between the yield (kg) of the grapevines in the images of the training set, and the number of pixels corresponding to the Grape class, computed by the classification methodology and image analysis. (**b**) Comparison between the actual values of yield (g) of the grapevines in the images of the validation set, and the predicted yield values calculated with the correlation equation in (a). Dotted line represents the 1:1 line.

The correlation and validation curves for the estimation of leaf area and grape yield using the classification methodology and image analysis are shown in Figures 8 and 9, respectively.

The actual leaf area on the grapevines’ images and the number of pixels corresponding to the Leaf class were found to be strongly correlated, following a linear relationship (y = 0.1712x + 0.1863) with coefficient of determination R^2^ = 0.78 at *p* < 0.001 (Figure 8(a)). When this function was used to predict the leaf area of another set of grapevine images (validation set), the correlation between the observed and predicted leaf areas was very close to the 1:1 line (y = 1.0598x + 0.0117) and the values of R^2^ = 0.81 at *p* < 0.001 and RMSE = 0.745 m^2^ (Figure 8(b)).

The differences in foliar density of the imaged vines, as interpreted as more or less number of leaf layers, may have impacted the estimation of the grapevine’s leaf area by image analysis. In this way, in very dense canopies (with several superimposed layers of leaves) the initial defoliation steps (Images I1 and I3) might not have caused a significant “disappearance” of leaves from the image, as expected, so that the vine remained fully covered with leaves, and only until the defoliation stage 3 was reached, the observed grapevine canopy area was drastically reduced. In other words, for very dense canopies, the removal of leaves in the very early steps did not always mean lower estimated leaf area by image analysis.

The images used in the present work corresponded to grapevines of medium to very dense canopies, in general, which seems to be the least favourable scenario for the estimation of leaf area by image analysis. However, the prediction of the leaf area from the model established by image analysis was very satisfactory, and it should be expected to perform better for grapevines with less dense canopies, as it is the case of low to moderate vigour vineyards.

Furthermore, a reliable and accurate estimation of the grapevine leaf area at several timings during the growing season may be of great usefulness to the grapegrower to monitor the vegetative growth of the plant, and identify symptoms of several abiotic and biotic stresses, such as water stress and diseases pressure, respectively, in a dynamic way. Likewise, this information may also help the grapegrower in taking canopy management decisions to improve the balance between vegetative and reproductive growth.

Regarding grape yield estimation, the correlation between the actual yield on the grapevines’ images and the number of pixels corresponding to the Grape class followed a linear relationship (y = 0.1787x + 0.611) with coefficient of determination R^2^ = 0.78 at *p* < 0.001 (Figure 9(a)).

When this curve was employed to predict the yield of the images of the validation set, the correlation between the observed and predicted yield values was also close to the 1:1 line (y = 0.8907x + 0.253), with a R^2^ = 0.73 at *p* = 0.002, and RMSE = 0.749 kg (Figure 9(b)).

In dense and very dense canopies, grape clusters are typically covered with leaves, preventing them from being exposed to the sun and also visible to the human or machine vision. This fact, which occurred in the images of the initial non-defoliated, non-thinned grapevines (I0) and also in images corresponding to the first defoliation step (I1 and I2), seems to have impacted the performance of the yield prediction by the image analysis methodology, as the coefficient of determination values for yield estimation were smaller than those for leaf area prediction.

Similarly to leaf area, the prediction of the grape yield from the model established by image analysis was satisfactory, and covered a broad range of grape exposure and visibility conditions, generated by the successive defoliation and grape thinning steps. When grapes in the canopy are partially covered by the leaves during maturation, and at harvest (especially in moderate to high vigour vineyards and in vineyards where defoliation was not performed or was only mildly performed) this method seems to be more applicative for leaf area estimation than yield. However, basal defoliation is a canopy management practice, widely conducted worldwide, between fruit-set and veraison, on one or two sides of the canopy, which is aimed at improving the fruit exposure for grape quality [15,17,44–47] and health purposes [48]. Since the visibility of the clusters is certainly increased after basal defoliation, the accuracy of the yield estimation by the classification methodology and image analysis presented in this work would significantly increase, hence allowing a very accurate yield prediction.

A truthful estimation of the potential grape yield soon after veraison is very valuable information not only for logistical purposes at harvest (*i.e.*, labour needs, winemaking capacity at the winery…) but also for economic reasons, especially when a wine producer has to buy grapes from other grapegrowers and suppliers, as the total grape yield of a given region or appellation area is an important driving force of the final grape price in a given vintage.

## 4. Conclusions

The methodology for canopy feature extraction and image analysis described in the present work has proved to be a useful and reliable tool for leaf area and yield assessment in the vineyard. It seems, though, more applicative to leaf estimation as grape visibility may be limited across the ripening period and harvest in non-defoliated, moderate to high vigour canopies. The setup proposed is simple, inexpensive and non-destructive for image-acquisition as only a commercial RGB camera is needed. The processing methodology has shown to be highly adaptable and robust to changes in illumination and in the distance to the targeted grapevine, which are two critical factors in machine vision applications under field conditions.

The classification methodology allowed discriminating seven different classes, corresponding to seven types of canopy features in the grapevines’ images, although only the Leaf and Grape classes were successfully calibrated and validated against real plant measurements. The classifier’s performance for the identification of leaves and grapes was very high and their effectiveness exceeded the 90% in both cases.

An accurate estimation of the grapevine leaf area and yield during the growing season by a fast and non-destructive method, such as the one described in this work, may provide very valuable information for the grape and wine industry for canopy management decisions, as well as for logistical and economical purposes, and can be further implemented for on-board analysis.

## Figures and Tables

**Table 1.** Description of the defoliation and de-fruiting steps during the session of image acquisition of each individual vine.

**Image #**	**Canopy manipulation event**	**Image**
I0	Initial stage of the vine (step 0)	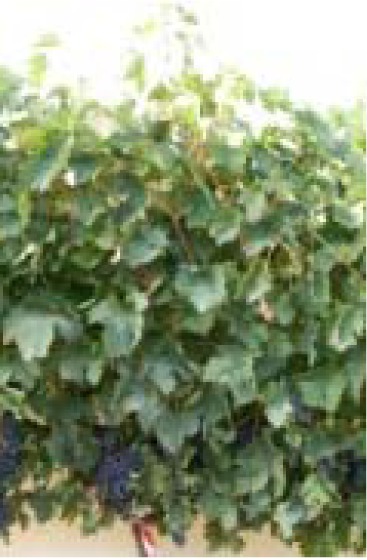
I1	Removal of the first 6 basal leaves per shoot (step 1)	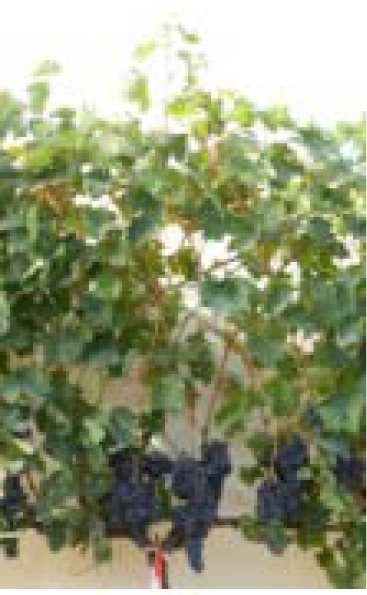
I2	Removal of one third of clusters (step 2)	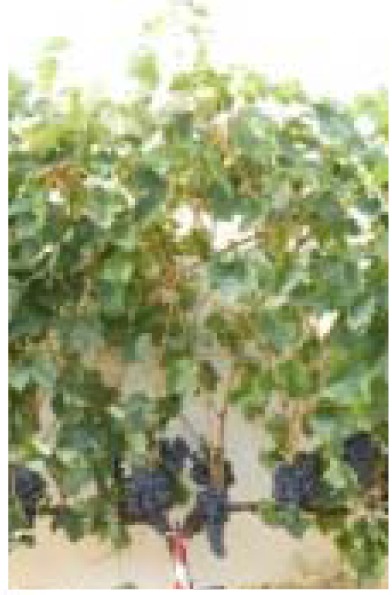
I3	Removal of additional 6 basal leaves per shoot (12 leaves removed in total) (step 3)	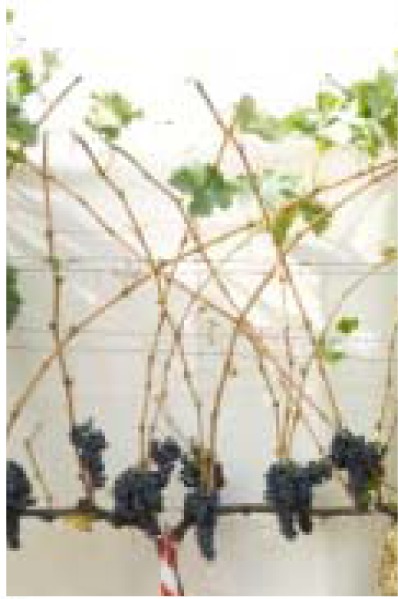
I4	Removal of one third of clusters (step 4)	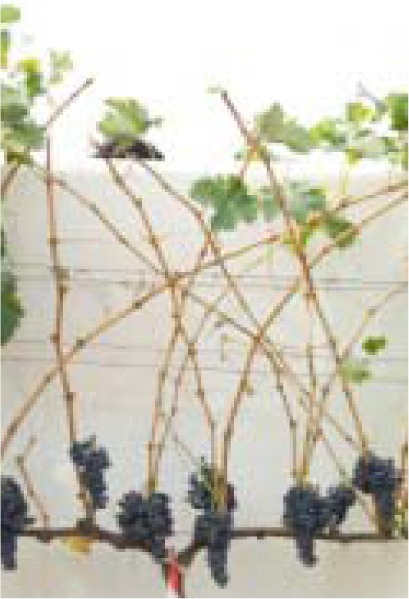
I5	Removal of remaining main leaves and laterals (step 5)	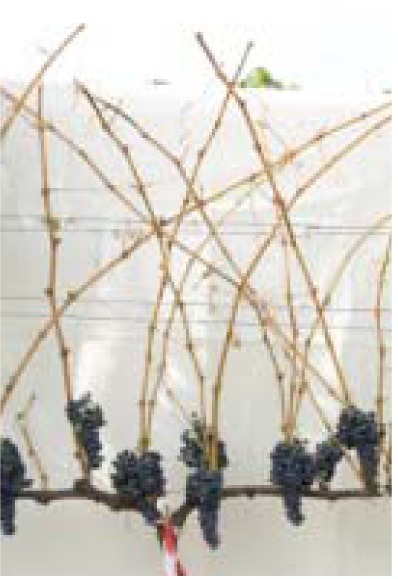
I6	Removal of all remaining clusters (step 6)	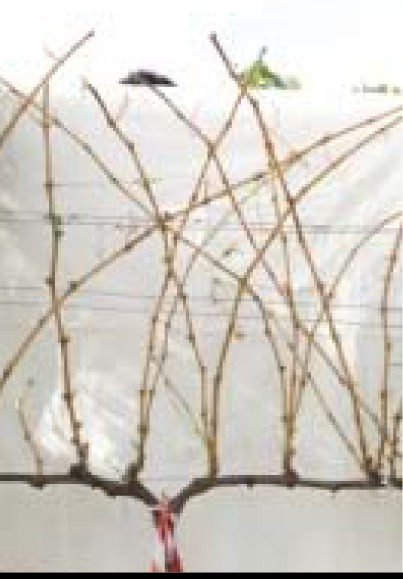

## References

[1] Dunn G.M., Martin S.R. (2004). Yield prediction from digital image analysis: A technique with potential for vineyard assessments prior to harvest. Aust. J. Grape Wine Res.

[2] Clingeleffer P.R., Krstic M. (2001). Final Report for Project CSH 96/1. Crop Development, Crop Estimation and Crop Control to Secure Quality and Production of Major Wine Grape Varieties: A National Approach.

[3] Wolpert J.A., Vilas E.P. (1992). Estimating vineyard yields: Introduction to a simple, two-step method. Am. J. Enol. Vitic.

[4] Dunstone R.J. (2002). Final Report for Project DNR 02/02. Winegrape Crop Forecasting Module.

[5] Dunn G.M., Martin S.R. (2003). The current status of crop forecasting in the australian wine industry. ASVO Seminar Series: Grapegrowing at the Edge.

[6] Martin S., Dunstone R., Dunn G. (2003). How to Forecast Wine Grape Deliveries Using Grape Forecaster Excel Workbook Version 7.

[7] Blom P.E., Tarara J.M. (2009). Trellis tension monitoring improves yield estimation in vineyards. HortScience.

[8] Smart R., Robinson M. (1991). Sunlight into the Wine. A Handbook for Winegrape Canopy Management.

[9] Bertamini M., Tardaguila J., Iacono F. (1994). Valutazione dell’equilibrio vegeto-produttivo e microclimatico del vigneto per l’ottimizzazione delle tecniche colturali a verde: Aspetti teorici e pratici. Rivista dell’Istituto Agrario San Michele all’Adige (Bolletino ISMA).

[10] Gray J.D., Gibson R.J., Coombe B.G., Giles L.C., Hancock T.W. (1994). Assessment of winegrape value in the vineyard—A preliminary, commercial survey. Aust. NZ Wine Ind. J.

[11] Carbonneau A. (1995). La surface foliaire exposee potentielle. Guide pour sa mesure. Prog. Agric. Vitic.

[12] Tardaguila J., Martines de Toda F. (2008). Assessment of tempranillo grapes quality in the vineyard by vitur score-sheet. J. Int. Sci. Vigne Vin.

[13] Kliewer W.M., Lider L.A. (1968). Influence of cluster exposure to sun on composition of thompson seedless fruit. Am. J. Enol. Vitic.

[14] Kliewer W.M. (1970). Effect of day temperature and light intensity on coloration of vitis vinifera l grapes. J. Am. Soc. Hort. Sci.

[15] Crippen D.D., Morrison J.C. (1986). The effects of sun exposure on the compositional development of cabernet sauvignon berries. Am. J. Enol. Vitic.

[16] Crippen D.D., Morrison J.C. (1986). The effects of sun exposure on the phenolic content of cabernet sauvignon berries during development. Am. J. Enol. Vitic.

[17] Reynolds A.G., Pool R.M., Mattick L.R. (1986). Influence of cluster exposure on fruit composition and wine quality of seyval blanc grapes. Vitis.

[18] Mabrouk H., Sinoquet H. (1998). Indices of light microclimate and canopy structure of grapevines determined by 3D digitising and image analysis, and their relationship to grape quality. Aust. J. Grape Wine Res.

[19] Smart R.E. (1985). Principles of grapevine canopy microclimate manipulation with implications for yield and quality. A review. Am. J. Enol. Vitic.

[20] Garrido M., Mendez V., Valero C., Correa C., Torre A., Barreiro P. Online dose optimization applied on tree volume through a laser device.

[21] Fleck S., van der Zande D., Schmidt M., Coppin P., Thies M., Spiecker B.K.H., Weinacker H. (2004). Reconstruction of tree structure from laser-scans and their use to predict physiological properties and processes in canopies. International Archives of Photogrammetry, Remote Sensing and Spatial Information Sciences.

[22] Moorthy I., Miller J.R., Berni J.A.J., Zarco-Tejada P.J., Qingmou L. Extracting tree crown properties from ground-based scanning laser data.

[23] Dey D., Mummert L., Sukthankar R. Classification of plant structures from uncalibrated image sequences.

[24] Correa C., Valero C., Barreiro P., Diago M.P., Tardaguila J. Feature extraction on vineyard by gustafson kessel fcm and k-means.

[25] Correa Farias C., Valero Ubierna C., Barreiro Elorza P. Characterization of vineyard’s canopy through fuzzy clustering and svm over color images.

[26] Meunkaewjinda A., Kumsawat P., Attakitmongcol K., Srikaew A. Grape leaf disease detection from color imagery using hybrid intelligent system.

[27] Berenstein R., Shahar O.B., Shapiro A., Edan Y. (2010). Grape clusters and foliage detection algorithms for autonomous selective vineyard sprayer. Intell. Serv. Robot.

[28] Braun T., Koch H., Strub O., Zolynski G., Berns K. Improving pesticide spray application in vineyards by automated analysis of the foliage distribution pattern in the leaf wall.

[29] Nuske S., Achar S., Bates T., Narasimhan S., Singh S. Yield estimation in vineyards by visual grape detection.

[30] Tardaguila J., de Toda F.M., Poni S., Diago M.P. (2010). Impact of early leaf removal on yield and fruit and wine composition of vitis vinifera L. Graciano and carignan. Am. J. Enol. Vitic.

[31] Tardaguila J., Herrero-Langreo A., Barreiro P., Valero C., Poni S., Diago M.P. Using rgb image analysis to assess the impact of early defoliation on the fruit zone.

[32] Kotsiantis S.B. (2007). Supervised machine learning: A review of classification techniques. Informatica.

[33] Tian L.F., Slaughter D.C. (1998). Environmentally adaptive segmentation algorithm for outdoor image segmentation. Comput. Electron. Agric.

[34] Bjurström H., Svensson J. (2002). Assessment of grapevine vigour using image processing.

[35] Chamelat R., Rosso E., Choksuriwong A., Rosenberger C., Laurent H., Bro P. Use of zernike moments for grape detection with image processing.

[36] Correa C., Moya A., Baguena E., Herrero A., Diago M.a.B. J., Tardaguilla J., Valero C., Barreiro P. Feature extraction of the vineyard canopies, using images acquired on-the-go (RGB and RGIR.

[37] Correa C., Valero C., Barreiro P., Diago M.P., Tardaguila J. A comparison of fuzzy clustering algorithms applied to feature extraction on vineyard.

[38] González D.P. (2010). Algoritmos de agrupamiento basados en densidad y validación de clusters.

[39] Son J., Inoue N., Yamashtia Y. (2010). Geometrically local isotropic independence and numerical analysis of the mahalanobis metric in vector space. Patt. Recog. Lett.

[40] McLachlan G.J. (1999). Mahalanobis distance. Reson. J. Sci. Educ.

[41] Al-Otum H.M. (2003). Morphological operators for color image processing based on mahalanobis distance measure. Opt. Eng.

[42] Herrero-Langreo A., Barreiro P., Diago M.P., Baluja J., Ochagavia H., Tardaguila J. Pixel classification through mahalanobis distance for identification of grapevine canopy elements on rgb images.

[43] Palliotti A., Cartechini A., Ferranti F. (2000). Morpho-anatomical and physiological characteristics of primary and lateral shoot leaves of cabernet franc and trebbiano toscano grapevines under two irradiance regimes. Am. J. Enol. Vitic.

[44] Percival D.C., Fisher K.H., Sullivan J.A. (1994). Use of fruit zone leaf removal with vitis vinifera l. Cv. Riesling grapevines. Ii. Effect on fruit composition, yield, and occurrence of bunch rot (botrytis cinerea pers.:Fr.). Am. J. Enol. Vitic.

[45] Reynolds A.G., Yerle S., Watson B., Price S.F., Wardle D.A. (1996). Fruit environment and crop level effects on pinot noir. III. Composition and descriptive analysis of oregon and british columbia wines. Am. J. Enol. Vitic.

[46] Bergqvist J., Dokoozlian N., Ebisuda N. (2001). Sunlight exposure and temperature effects on berry growth and composition of cabernet sauvignon and grenache in the central san joaquin valley of california. Am. J. Enol. Vitic.

[47] Kliewer W.M., Dokoozlian N.K. (2005). Leaf area/crop weight ratios of grapevines: Influence on fruit composition and wine quality. Am. J. Enol. Vitic.

[48] Bledsoe A.M., Kliewer W.M., Marois J.J. (1988). Effects of timing and severity of leaf removal on yield and fruit composition of sauvignon blanc grapevines. Am. J. Enol. Vitic.

